# Sustainable Enhanced Oil Recovery Fluid Based on Synergic Effects of Cationic, Anionic, and Nonionic Surfactants in Low Salinity: SLS; QA; and SDBS

**DOI:** 10.1021/acsomega.4c10089

**Published:** 2025-02-22

**Authors:** Cristina M. Quintella, Pamela Dias Rodrigues, Samira Abdallah Hanna, Jorge Luis Nicoleti, Edgard Bacic Carvalho, Ana Claudia Gondim de Medeiros, Elias Ramos-de-Souza, Elias Silva dos Santos, Anaís Couto Vasconcelos, Juliana Dias de Moura, Eloísa Vitória Almeida de Oliveira Santos, Matheus de Oliveira Gama, Vitor Cerqueira Morais

**Affiliations:** † Chemistry Institute, Federal University of Bahia, Ondina Campus, Salvador 40.170-115, Bahia, Brazil; ‡ Energy and Environment Center, Federal University of Bahia, Ondina Campus, Salvador 40.170-115, Bahia, Brazil; § Institute of Health Sciences, Federal University of Bahia, Canela Campus, Salvador 40.231-300, Bahia, Brazil; ∥ Federal Institute of Education, Science and Technology of Bahia, Salvador Campus, Salvador 40.301-015, Bahia, Brazil; ⊥ Mosaico Fluido Pesquisa e Inovação Ltda, Rua Ewerton Visco, No. 324, Ed. Holding Empresarial, Caminho das Árvores, salas 201/202, Salvador 41820-022, Bahia, Brazil

## Abstract

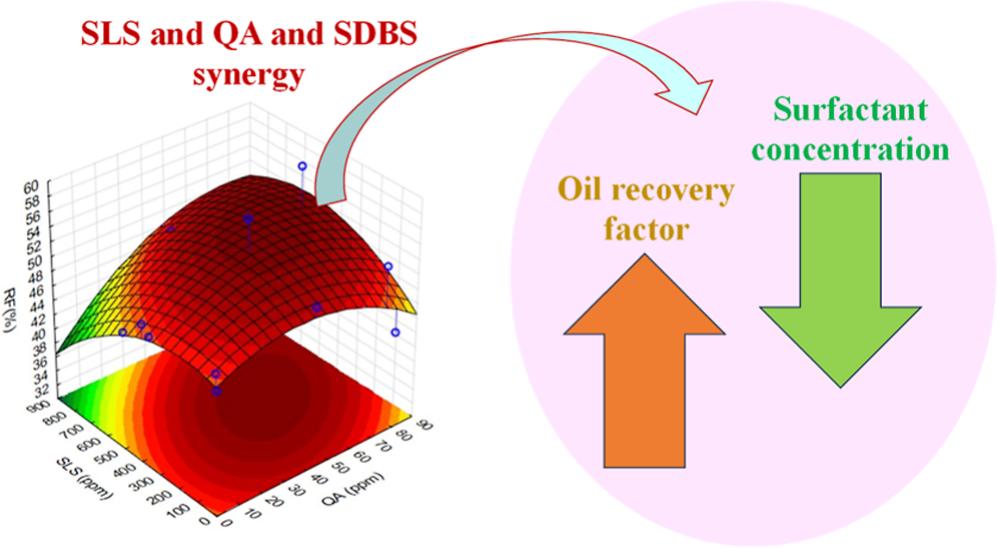

In the context of the energy transition, petroleum will remain a critical resource for several decades. Enhanced oil recovery (EOR) offers a method for optimizing its production while advancing the United Nations Sustainable Development Goal 12 (SDG 12) and the 2030 Agenda, which emphasize reducing the environmental impact and addressing community concerns. Pre-salt limestone reservoirs, situated far offshore, require customized enhanced oil recovery (EOR) strategies. Although low salinity water has recently gained attention for these reservoirs, further investigation is required to integrate environmentally low-impact surfactants and to minimize their concentrations while maintaining high recovery factors (RFs). Low salinity water formulations containing small concentrations of surfactants were injected into limestone core samples with oil from presalt reservoirs under reservoir conditions. In total, 21 linear formulations and 16 multivariate formulations experimentally designed using Doehlert matrix as the chemometric tool were prepared by combining the nonionic surfactant sodium dodecylbenzenesulfonate (SDBS), the anionic surfactant sodium lauryl sulfate (SLS), and the cationic surfactant tetraethylammonium chloride (QA) at concentration levels above, below, and on their respective critical micelle concentrations (CMCs). The produced water was analyzed using X-ray fluorescence, with spectra evaluated via principal component analysis (PCA) and partial least squares (PLS). Theoretical simulations were also performed with molecular dynamics applied to the two top-performing results. The traditional linear study, varying individual surfactant concentrations, yielded an RF increase of up to 12.8%. However, the covariation approach further improved the results, achieving an RF of 16%, primarily due to the synergistic effects of the cationic and anionic surfactants, which mobilized both bulk oil and surface oil. The balance among micellar SLS, micellar SDBS, and nonmicellar QA proved essential. Theoretical simulations supported these experimental findings, indicating no direct interaction between the surfactants, increased interfacial tension, SLS migration to the surface, and QA retention in the bulk phase.

## 1 Introduction

In relation to the responsible transition of energy sources, it is imperative to acknowledge that the planet will maintain a requirement for petroleum over the course of several decades. Enhanced oil recovery (EOR) methods offer a means of intelligently managing its extraction while aligning with the objectives outlined in the United Nations Sustainable Development Goal 12 (SDG 12) and the 2030 Agenda. These initiatives require a sustained decrease in environmental repercussions and address community concerns. For instance, the pre-salt reserves, located far from continents, mandate tailored EOR approaches specifically designed for limestone reservoirs.

However, it is imperative to minimize their environmental footprint. Consequently, ongoing efforts are focused on researching EOR formulations that significantly reduce environmental footprints.^1^

As oil reserves deplete and global energy demand rises, there is a pressing requirement for research aimed at uncovering new insights into the fundamental aspects of fluids utilized in oil recovery and their relationship with fluid salinity, influencing this pivotal process.

EOR with low-salinity solutions (smart waters) emerged approximately a decade ago following its recognition for the potential to increase the recovery factor (RF), being a method suitable to the pre-salt.^2−6^ This emerging technique started in 2008, both in published articles and in filled patents.^7,8^ Several additives may be added to smart water in order to improve its recovery efficiency, among them, surfactants.^9,10^

Despite the recent emergence of low salinity waters as a promising technique for limestone reservoirs (chemically different from sandstone reservoirs), there exists a clear knowledge gap regarding the incorporation of surfactants with minimal environmental impact.

EOR in limestone differs from that in sandstone due to the inherent oil-wettability of carbonate formations, requiring a transition to water-wettability for effective oil displacement. The utilization of surfactants emerges as a viable solution in achieving this alteration in wettability and facilitating oil removal from carbonate reservoirs.^11,12^

Surfactants are extensively used in the petroleum industry for their ability to modify the water–oil interface, thereby enhancing the hydrocarbon displacement efficiency and ultimately increasing well recovery rates. They are employed in various petroleum processes, including well drilling, cleaning agents, demulsifiers, lubricants, foaming agents, detergents, solubilizers, and dispersants.^13^ Commercially available surfactants, commonly utilized in the industry, contribute to achieving satisfactory oil recovery rates, despite the challenge of high costs when used at a large scale.^14^ They can yield an additional 9.11% of oil recovery.^15^

The use of salts in injection waters significantly impacts surfactant behaviors, altering their critical micelle concentration (CMC) and affecting the intermolecular forces among formation water, oil, and the calcareous surface.^16^ It is essential to review surfactant behaviors when exposed to salt presence and the impact of specific ion concentrations on the performance of low-salinity water injection.

Combining surfactants with low-salinity water flooding represents a highly effective approach in advanced oil recovery, as it decreases oil–water interfacial tension and alters rock wettability.^17^ However, the interfacial tension reduction between crude oil and brine is strongly influenced by the type and concentration of ions present in the brine. Specific salinity ranges enable interfacial tension to reach ultralow values, with NaCl commonly preferred due to its higher concentration in produced water from oil reservoirs and its significant effect on the properties of surfactants used in advanced oil recovery.^18,19^

In recent years, the number of patents in the field of EOR involving the association of surfactants and NaCl has surpassed the number of research articles, pointing to a technological challenge.^20^

Cationic surfactants used in EOR, typically comprise molecules with quaternary nitrogen bases, although often more expensive in bulk, become appealing when coupled with low salinity water technology as small concentrations are needed. This attractiveness arises because they possess a charge akin to the surfaces of carbonate minerals, thereby potentially modifying the wettability of carbonates to a water-wet state.^12^

The use of doubly charged ions in EOR for carbonate reservoirs, in conjunction with the cationic surfactant cetyltrimethylammonium bromide (CTAB), has shown that small amounts of CTAB reduce interfacial tension, alter wettability, increase pore pressure, and enhance oil recovery.^21^ Yet, when utilized in asphaltene-rich oil, electrostatic interactions between the positively charged head of the surfactant and the negatively charged carboxylate result in adsorption of the cationic surfactant onto the oil-wet surface. This alters the rock from being oil-wet to water-wet upon removal of the carboxylate group from the rock surface, forming an ion pair, but could potentially lead to a decrease in cationic surfactant concentration, oil snap-off, asphaltene deposition, and the risk of pore plugging.^22^

QA is considered to be one of the smaller cationic surfactants. It is used as a pharmacological research agent that blocks selective potassium channels, as a basic laboratory chemical reagent, and as a low-residue bactericide in hydrofracking.^23^ It is not toxic at concentrations inferior to 2630 mg/kg (2,630 ppm).^24^ For EOR, QA is not usually used, although there are some studies of another quaternary ammonium salt, CTAB.

Anionic surfactants for EOR, such as sulfonated compounds, alcohol propoxylate sulfates, and internal olefin sulfonates, demonstrate high effectiveness in reducing the interfacial tension between the injection water and oil. They exhibit low adsorption on carbonate and calcite surfaces, thereby enhancing water injection performance.^13,25^

The widely recognized anionic SLS finds extensive use in cleaning products due to its multifunctional properties as an emulsifier, foaming agent, and surface wetting agent, enabling it to emulsify or solubilize oils and suspend soil for easy rinsing. According to the American Cleaning Institute,^26^ SLS is considered safe for use in cleaning products, with the U.S. Environmental Protection Agency (EPA) setting a tolerance limit for food residues at 350 ppm. This surfactant has been employed in hydraulic fracturing fluids, with well-established safety regulations within the crude oil industry.^27^ SLS demonstrates rapid biodegradability under both aerobic and anaerobic conditions, hence it does not persist in the environment.^28^ However, a recent review noted the absence of conclusive results regarding the environmental impact of SLS.^29^ In EOR applications, SLS has been used as a viscosity reducer for extracting highly viscous oils, typically in relatively high concentrations.^30^

There is controversy in the literature concerning the comparison of effectiveness between cationic and anionic surfactants in carbonate reservoirs. Anionic surfactants change the wettability of the calcite surface to intermediate/water-wet condition as well or better than the cationic surfactant DTAB for a West Texas crude oil.^12^ Cationic surfactants can adsorb much better than other types of surfactants (nonionic and anionic) on the oil–water interface and reduce the IFT value of the oil-wet carbonate core.^25^ Cationic surfactants generally outperform anionic ones in altering wettability due to stronger ionic interactions.^31−33^

Laboratory experiments conducted on carbonate cores indicated that the addition of sodium dodecylbenzenesulfonate (SDBS), a nonionic surfactant, in low salinity waters at a concentration of about 0.5% by weight resulted in only a modest 2% enhancement in RF.^34^

The nonionic surfactant SDBS finds widespread applications across various industries including household care products, cleaning agents, detergent powders, dishwashing liquids, toilet cleaners, industrial detergents, oil drilling, gas, rubber, NBR, leather, gloves, biocide, and pesticide sectors. It exhibits rapid biodegradation under both aerobic and anaerobic conditions in water, sediments, and soil.^35^ While SDBS has shown promise in EOR, its application has primarily been limited to conditions lacking salt presence.^36^

Esfandiarian et al.^37^ obtained pendant drops of low salinity water with either the cationic CTAB or of the anionic SDBS and found that higher concentrations (above CMC) were necessary to significatively lower the interfacial tension and potentially increase RF.

Synergistic effects between different surfactants play a crucial role in improving the oil recovery efficiency. When two or more surfactants, such as cationic and anionic or nonionic and ionic, are combined, they can form mixed micelles or interact to enhance individual properties, such as reducing interfacial tension, which is vital for mobilizing trapped oil in porous media. Mixed surfactants may also stabilize micellar structures better than single surfactants.^38^ For example, in sandstone reservoirs, combining surfactants such as SDS (anionic) and CTAB (cationic) has been shown to achieve a greater shift from oil-wet to water-wet conditions than using either surfactant alone, a critical factor for enhanced oil displacement. This wettability change is essential for enhanced oil displacement. Mixed surfactant systems exhibit improved thermal and salinity stability, overcoming challenges typically observed with single surfactants, such as mitigating precipitation problems in high salinity environments.^39^ While significant research exists on synergistic surfactants in sandstone formations, studies on carbonate reservoirs remain relatively scarce due to their complex oil-wet nature and heterogeneity. However, emerging research suggests that the synergistic use of cationic surfactants with nonionic surfactants or even biosurfactants can effectively modify wettability and improve oil recovery in carbonates.^38^

This study aims to assess potential increases in EOR RF under limestone reservoir conditions by exploring synergistic interactions among various surfactant types within low-saline injection water. Emphasis is placed on using minimal surfactant concentrations to reduce environmental impacts. Through a multivariate experimental design, this study seeks to identify concentrations, where synergies among cationic, anionic, and nonionic surfactants enhance RF while maintaining environmentally conscious surfactant levels.

## 2 Methods

This section details materials, methods, and procedures, emphasizing modifications and adaptations made to previously published setups.^40−42^

### 2.1 Pre-Salt Oil and Limestone Cores

The dead oil utilized was the condensed fraction from the Jupiter/RJ pre-salt field, possessing a total acid number (TAN) of 1.01 mgKOH/g (classified as acidic, exceeding 0.5 mg_KOH_/g), a viscosity of 2.04 cP at 65 °C, a density of 0.88 g/mL, and an API gravity of 28. SARA analysis revealed a composition of 50.7% saturates, 18.8% aromatics, and 17.8% resins, with the remaining 12.7% attributed to volatile losses and difficulties in filtering the asphaltene fraction falling within the permissible limit of the applied standard.

The limestone cores, from Mt. Gambier and obtained from Kokurec Inc., had a nominal permeability of 1000 mD, a diameter of 3.8 cm, and a length of 7.0 cm. Surface characterization conducted using scanning electron microscopy unveiled a broad spectrum of pore diameters, spanning from approximately 100 to 500 μm.

The cores were deaerated by immersing each one in synthetic formation water under vacuum conditions (1 × 10^–3^ Torr using an Edwards model RV5 vacuum pump) for at least 5 h until no gas release was observed. The pore volume of each core was calculated by determining the mass difference between the dry core and the saturated core, divided by the specific mass of the synthetic formation water.

### 2.2 Low Salinity Water Solutions and Synthetic Formation Water

The synthetic formation water used was prepared by mixing deionized water with PA-grade purity salts: 200,000 ppm of NaCl and 30,000 ppm of CaCl_2_. It displayed a viscosity of 0.72 cP at 65 °C, a density of 1.15 g/mL, and a total dissolved solid (TDS) content of 230,000 ppm. Ionic concentrations included Na^+^ (78,700 ppm), Ca^2+^ (10,838 ppm), and Cl^–^ (140,462 ppm).

The ideal concentration of low salinity water for injection was established based on Quintella et al.^42^ at 18,466 ppm of NaCl, 1923 ppm of CaCl_2_, and 608 ppm of NaHCO_3_.

Despite surfactants’ well-established capability to improve the RF, their pronounced interaction with the human body and ecosystems emphasizes the necessity to prioritize studies focusing on low environmental impact. Prior to conducting laboratory experiments, a comprehensive scientific and technological review of surfactant utilization in EOR was performed to select suitable candidates.^20^ Subsequently, surfactants demonstrating lower environmental impact were chosen for the experiments.

Three surfactants were selected, namely, the nonionic sodium dodecylbenzenesulfonate, SDBS (Cas: 25155-30-0), the anionic sodium lauryl sulfate, SLS (Cas: 151-21-3), and the cationic tetraethylammonium chloride, QA (Cas: 56-34-8).

**Figure 1:**
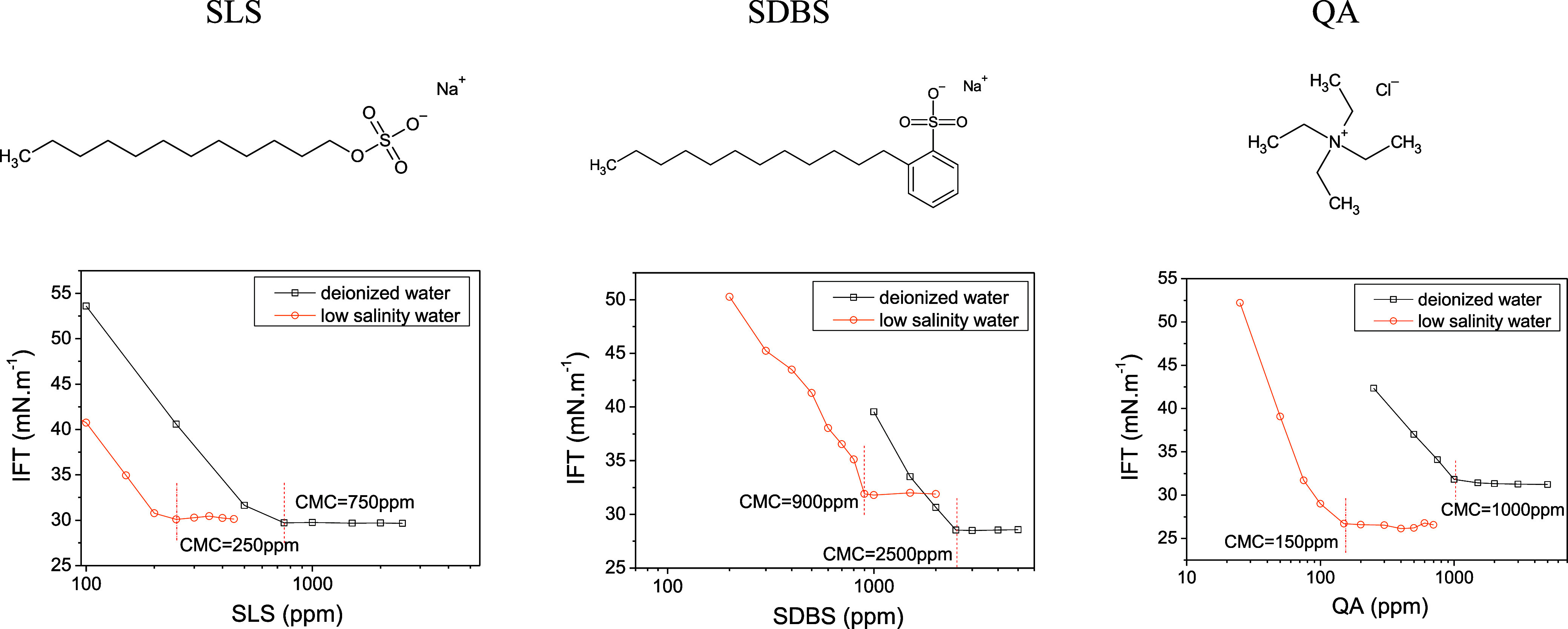
Top—Molecular representations of surfactants, drawn by the authors using ACD/ChemSketch. Bottom—Curves depicting critical micellar concentration (CMC) determinations of surfactants in deionized water (in black) and within a low salinity water solution containing 18,466 ppm of NaCl, 1923 ppm of CaCl_2_, and 608 ppm of NaHCO_3_: SDBS, SLS, and QA (in red).

Figure 1 illustrates the molecular representations of the surfactants and shows their CMCs, determined via pending drop contact angle measurements, within a low salinity water environment previously optimized^42^ for the three salts,^43−45^ comprising 18,466 ppm of NaCl, 1923 ppm of CaCl_2_, and 608 ppm of NaHCO_3_.

### 2.3 Experimental Designs

Two experimental design strategies were employed. The first involved a traditional approach, assessing the impact on RF by linearly varying the concentration of each surfactant, independently. The second strategy utilized a covariant approach aimed at evaluating the potential synergistic effects among the three surfactants.

In the first strategy, the concentrations of the surfactants were selected based on their CMC in the low salinity water solution. The chosen concentrations included values above, equal to, and below the experimentally determined CMC. A total of 21 EOR tests were conducted using this approach.

The second strategy employed the covariant factorial design model Doehlert Matrix,^46^ using the best surfactant concentrations obtained from the linear strategy as variables. TIBCO Statistica Ultimate software was utilized to determine concentration levels, create 3D response surfaces, model specific variables, and perform data analysis. Sixteen planned EOR injection tests were conducted, covering 15 different combinations of surfactant concentrations along with a central point repetition. Subsequently, an additional 17th EOR test was performed upon the model revealing two maxima.

### 2.4 Core Holder Displacement Tests

**Figure 2:**
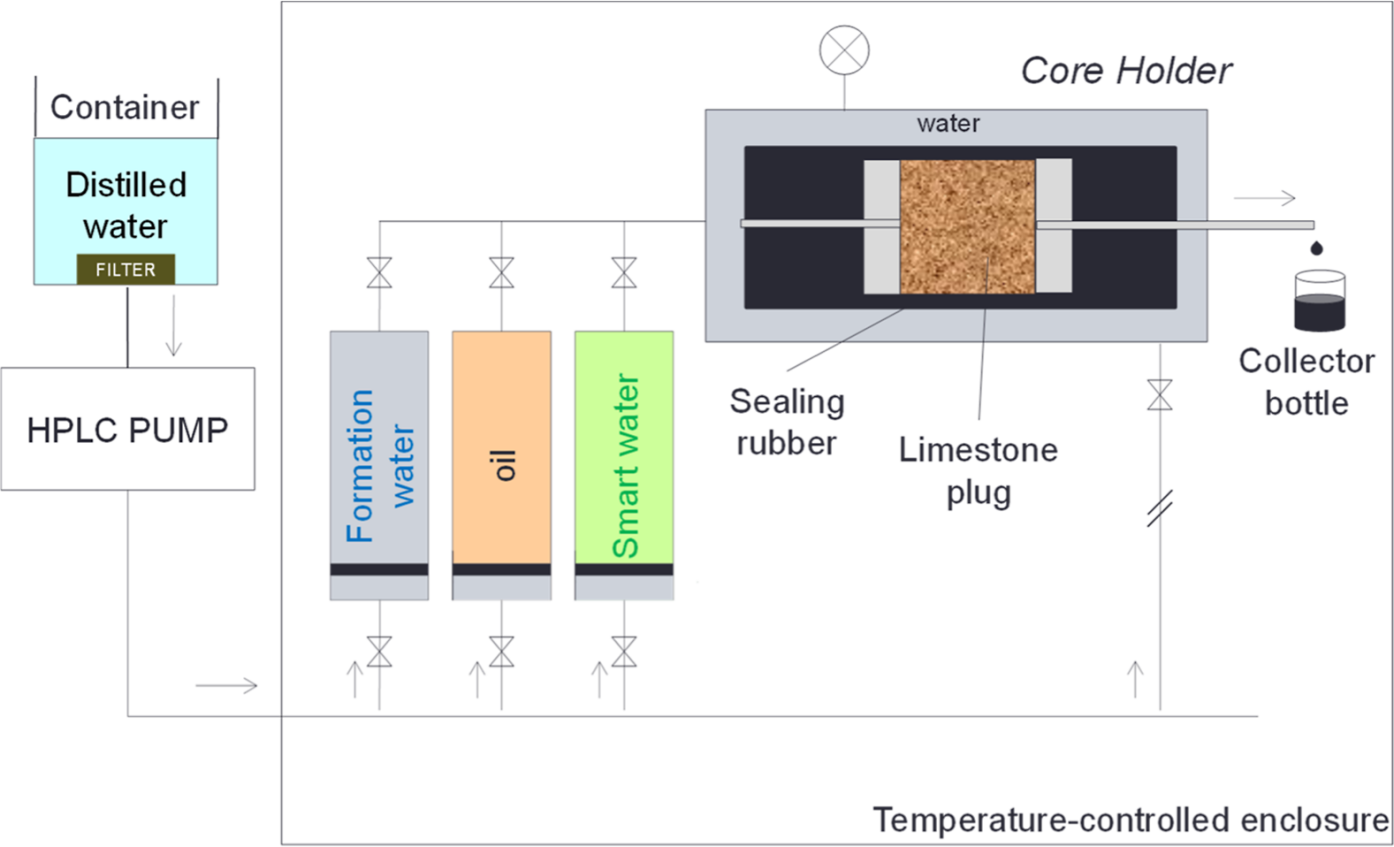
Schematic representation of the core holder system.

EOR displacement tests were conducted on saturated limestone core plugs using a core holder system within a temperature-controlled chamber, following prior established protocols^40−42^ with minor adaptations for early injection mode. An overview of the core holder system setup is presented in Figure 2.

The Jasco PU-4087 HPLC pump supplies the entire system with a constant flow rate of 0.8 mL min^–1^. The injection fluids are stored in three containers, each connected to the core plug holder through independent valves.

A confining pressure of 300 psi was employed to seal the lateral surface of the core plug, effectively preventing liquid flow between the core plug and the rubber sleeve. The tests were conducted at a temperature of 65 ± 2 °C, and for each displacement test, a new saturated plug was utilized.

To ensure core saturation, one porous volume of synthetic formation water was passed through the plug. Following this, two porous volumes of oil were injected to attain water residual saturation, which terminated the process when no water was observed at the outlet of the core plug holder. Subsequently, seven porous volumes of low salinity water were injected as a standard laboratory procedure, even if oil production ceased earlier, to ensure consistent oil collection across all experiments.

At intervals of every half-porous volume injected during the low salinity water injection runs, the collected fluids were accumulated in pre-weighed flasks, facilitating a subsequent mass balance assessment. Upon completion of each injection test, the produced fluids, comprising the aqueous and oil fractions, were separated and individually weighed. The aqueous fraction was refrigerated for storage, while the oil fraction was stored in sealed containers at room temperature.

The entire system undergoes a cleaning and reset procedure after each EOR injection test.

### 2.5 Samples Characterization

The injected and produced fluids underwent comprehensive analysis, encompassing rheology, pH measurement, conductivity measurement, molecular fluorescence spectroscopy (3D fingerprints), and X-ray fluorescence.

The pH measurements of the samples were conducted using an ASTRA PHS-3E pH meter, while the conductivity was determined using a Tecnal TEC-4MP conductivity meter. The pH of the injected low salinity waters ranged from 7.2 to 8.4, and their conductivity ranged from 37.8 to 48.5 mS cm^–1^. Both parameters exhibited sensitivity to the concentrations of surfactants, as anticipated. The pH of the produced water, after two porous volumes were injected, consistently fell within the range of 5.24 to 8.9 and its conductivity ranged from 18.7 to 255.9 t mS cm^–1^.

Oil, low salinity water, and produced fluids were rheologically characterized using an Anton-Paar Physica MCR 501 rheometer at 25 and 65 °C, with shear rates from 1 s^–1^ to 100 s^–1^. Temperature-dependent viscosity data were obtained by ramping the temperature from 40 to 80 °C while maintaining a constant shear rate of 7 s^–1^.

Oil fingerprints were obtained by diluting 0.02 g of oil in 20 mL of hexane PA, analyzing it in a PerkinElmer LS55 spectrofluorimeter. Ten scans were averaged, spanning excitation wavelengths of 250–450 nm and emission wavelengths of 300–900 at 1200 nm/min. This generated 3D fingerprints.

X-ray fluorescence analysis of the produced water was performed using a Shimadzu EDX900 spectrometer with a 10 mm collimator, air atmosphere, 50 kV, 1000 μmA, and 100 s irradiation time in QualiQuant mode with Kα acquisition.

Covariant analysis utilized PCA and its PLS modeling.

### 2.6 Molecular Simulation

To gain a clearer understanding of the role of surfactants, a molecular dynamics simulation was conducted, representing the Brazilian pre-salt conditions. The simulation model was constructed for a three-phase system enclosed in a rectangular box of 150 × 5 × 12 nm^3^, consisting of 8000 calcite (CaCO_3_) molecules to mimic the common rock found in Brazilian pre-salt reservoirs. Directly above this, a liquid layer (brine) containing 122,381 TIP 3p water molecules with the ionic concentration was added. Additionally, 400 H^+^ ions, derived from the deprotonation of naphthenic acid (ID7135, SpiderChem), were included in the synthetic brine. An oil layer was placed above the brine, composed of 400 molecules of naphthenic acid representing the polar component and 8000 molecules of *n*-decane (ID14840, SpiderChem) representing the nonpolar component.

The rock’s total surface charge was adjusted to 0 e.nm^2^, optimized by using the steepest descent algorithm with atom positions restricted with small force constant in all directions (1000, 1000, 1000) to fix them in space. The system was equilibrated for 4 ns by NPT ensemble to achieve an approximate density of 2.270 g/cm^3^, with a thickness of approximately 0.59 nm. Surfactant molecules, including SLS (CID 3423265), QA (CID 5946), and SBDS (CID 4289524), were obtained from the PubChem database and introduced into the brine. The topology of the molecules was generated using the ACPYPE web server.^47^ To mimic reservoir thermodynamic conditions, the V-rescale method^48^ was used to maintain the temperature at 338 K with a time constant of 0.1 ps, while the Parrinello–Rahman barostat^49^ was applied at 558 bar with a time constant of 5 ps to ensure constant pressure. The system was simulated for 200 ns using the OPLS force field in GROMACS version 2021.3.^50^

Interfacial tension was used to determine the ability of surfactants to enhance the RF, using the expression
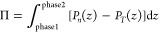
1where *P*_*n*_ is the normal pressure component, and *P*_*T*_ is the sum of the tangential components in the *x* and *y* directions.

## 3 Results and Discussion

Initially, the RF for deionized water and the RF of the optimized low salinity water were obtained and measured, resulting in values of 41.4% and 48.3%, respectively. These values served as baselines for assessing the RF increments.

Table 1 shows the RFs obtained with the first strategy of linear variation of the surfactant’s concentration for SLS, SDBS, and QA within the saline low salinity water solutions used as injection fluids.

**Table 1:** Linear Strategy for Low Salinity Water Formulations Showing the Concentrations of Each Surfactant Within the Saline Low Salinity Water (18,466 ppm of NaCl, 1923 ppm of CaCl_2_, and 608 ppm of NaHCO_3_) and the Recovery Factors (RFs) Obtained: SDBS, SLS, and QA

QA (ppm)	RF (%)	SLS (ppm)	RF (%)	SDBS (ppm)	RF (%)
deionized water	41.4	deionized water	41.4	deionized water	41.4
low salinity water	48.3	low salinity water	48.3	low salinity water	48.3
43	54.2	108	46.5	263	49.8
75	42.1	150	50.4	450	53.3
150^a^	50.8	250^a^	48.3	900^a^	46.2
150^a^	48.9	250^a^	47.7	900^a^	49.3
225	50.2	350	52.1	1350	51.1
256	48.8	391	49.1	1536	49.7

a Critical micellar concentration (CMC) concentration.

It is possible to observe that there is no simple distinct trend when the concentrations of either the cationic or the anionic surfactant are independently increased, which could explain the conflicting findings in the literature regarding the effectiveness of each of them. For instance, for carbonate reservoirs, Seethepalli et al.^12^ found that anionic surfactants were more effective than cationic surfactants, whereas Sofla et al.^25^ reported that cationic surfactants outperformed anionic surfactants.

Additionally, cationic surfactants are shown to contribute more significantly to the enhancement of RF compared to anionic SLS or nonionic SDBS. This effect is primarily attributed to the reduction of interfacial tension (IFT) at the limestone surface, as reported by Sofla et al.^25^

Using low salinity water containing QA resulted in an RF increase of up to 12.8% compared to deionized water when QA was in its nonmicellar form. However, in the micellar configuration, the increase in RF was only up to 9.4%. Interestingly, the concentrations reported by Esfandiarian et al.,^37^ determined through pendant drop experiments, suggested a requirement for significantly higher concentrations than those shown in Table 1—well above their critical micelle concentrations (CMCs) and the limits for safe environmental usage.

The addition of the anionic surfactant SLS to low-salinity water increased the RF by 10.7% compared to deionized water. It is interesting to note that below the CMC, when the concentration of molecules in the micellar conformation increases, there seems to be a tendency for an increase in the RF. However, when reaching the CMC, initially, the smaller-sized micellar form causes the RF to decrease. Subsequently, slightly above the micellar concentration, as the number of micelles increases, there is an increase in the RF. Then, as the micelle size increases with the concentration of SLS, the effect on the RF becomes smaller.

The nonionic surfactant SDBS increased the RF by 11.9% compared to deionized water and exhibited a 5% increase compared to low salinity water, reaching 53.3% at concentrations slightly lower than its CMC. Hence, the most favorable outcomes seem to emerge in the absence of micelles within the injection solution. These findings contradict the pending drop results reported by Esfandiarian et al.,^37^ which indicated concentrations well above the CMC. They also differ from the results of Imanivarnosfaderani et al.,^34^ as their concentrations were clearly higher than those detailed in Table 1. Given the objective of minimizing surfactant usage and potential environmental impacts, no high concentrations of surfactants were employed in this study.

Table 2 shows the covariant strategy for low salinity water formulations, using the best concentrations from the linear strategy.

**Table 2:** Covariant Strategy for Low Salinity Water Formulations Showing the Concentrations of Each Surfactant Within the Saline Low Salinity Water (18,466 ppm of NaCl, 1923 ppm of CaCl_2_ e 608 ppm of NaHCO_3_) and the Recovery Factors (RFs) Obtained: SDBS, SLS, and QA

QA (ppm)	SLS (ppm)	SDBS (ppm)	RF (%)
deionized water			41.4
low salinity water			48.3
0	0	800	49.3
0	350	450	48.2
0	500	0	49.1
0	500	800	46.2
43	0	450	51.9
43	350	0	58.0
43	350	1123	53.3
43	350	450	52.0
43	350	450	52.9
43	770	450	49.9
80	0	0	53.0
80	0	800	43.4
80	500	0	58.1
80	500	800	51.8
110	350	450	53.9

**Figure 3:**
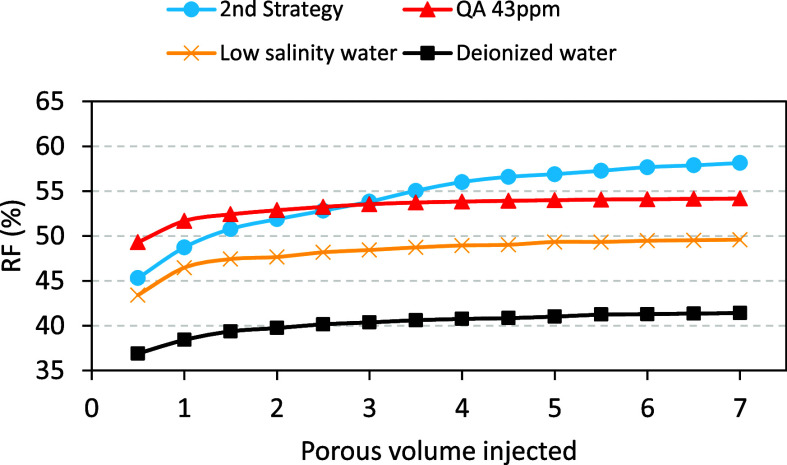
FR as a function of the injected porous volume for deionized water (squares), low salinity water (crosses), low salinity water with 43 ppm of QA (triangles), and low salinity water with 350 ppm of SLS, zero SDBS, and 43 ppm of QA (circles).

Figure 3 shows examples of the RF as a function of the injected porous volume for deionized water, low salinity water, low salinity water with 43 ppm of QA, and low salinity water with 350 ppm of SLS, zero SDBS, and 43 ppm of QA. It is possible to clearly observe the increase in recovery due to the synergistic effects of surfactants, despite their relatively low concentrations. Furthermore, traditional recovery methods do not lead to a significant increase in the RF after three injected pore volumes, while the combination of surfactants enables the RF to continue increasing until approximately six pore volumes.

In this covariant strategy, the RF increased by 16.7% compared to deionized water, 9.8% in relation to low salinity water, and 3.9% compared to the linear strategy in Table 1. The highest RFs were attained with the nonmicellar form of the cationic surfactant QA (43 and 80 ppm), the anionic SLS in its micellar configuration (350 and 500 ppm), and the absence of the nonionic surfactant SDBS.

**Figure 4:**
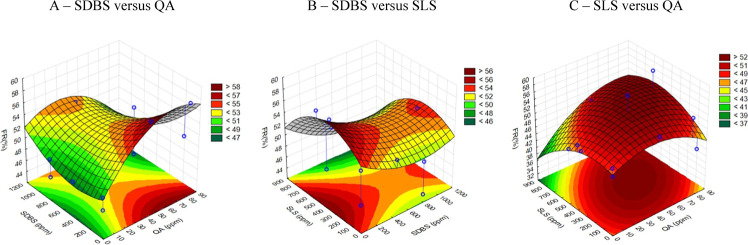
Surfaces of the EOR oil recovery factor (RF) with the low salinity water and the three surfactants: (A) SDBS (zero ppm to 1200 ppm) versus QA (zero ppm to 90 ppm) with SLS constant at 350 ppm; (B) SDBS (zero ppm to 1200 ppm) versus SLS (zero ppm to 900 ppm) with QA constant at 80 ppm; and (C) SLS (zero ppm to 900 ppm) versus QA (zero ppm to 90 ppm) with SDBS constant at 450 ppm.

The application of statistical analyses using Statistica software allowed for the modeling of results presented in Table 2, the evaluation of method reliability, and the assessment of the impact of surfactant concentration variations on the injection water formulations used during displacement tests. The obtained correlation coefficient was *R*^2^ = 0.945, with a residual error of 0.4%, confirming its reliability. Figure 4 illustrates the mathematically modeled RF surfaces using a non-factorial central composite design, with two variables varying at a time, while the third is kept constant at its central point value.

In the relationship between SDBS and QA (Figure 4A), a saddle-shaped surface can be observed, where the RF is maximized with moderate values of QA and higher or lower concentrations of SDBS.

Considering SDBS versus SLS (Figure 4B), the RF also exhibits a saddle-shaped surface with the regions of maxima corresponding to SDBS concentrations at the extremes (once again) and with moderate concentrations of SLS.

The mathematical RF surface for SLS versus QA reveals a maximum at moderate values of both surfactants.

The saddle-shaped surfaces indicate that two concentrations of SDBS maximize the RF: very low concentrations and those higher than those tested. The analysis recommended a formulation that achieved the highest RF at 350 ppm of SLS, 43.4 ppm of QA, and 1003 ppm of SDBS in its micellar form. An EOR displacement test was conducted with this concentration, resulting in an RF of 58.8%. Considering both the environmental impact and the experimental margin of error of 1%, the decision was made not to pursue the option of increasing the SDBS concentration.

**Figure 5:**
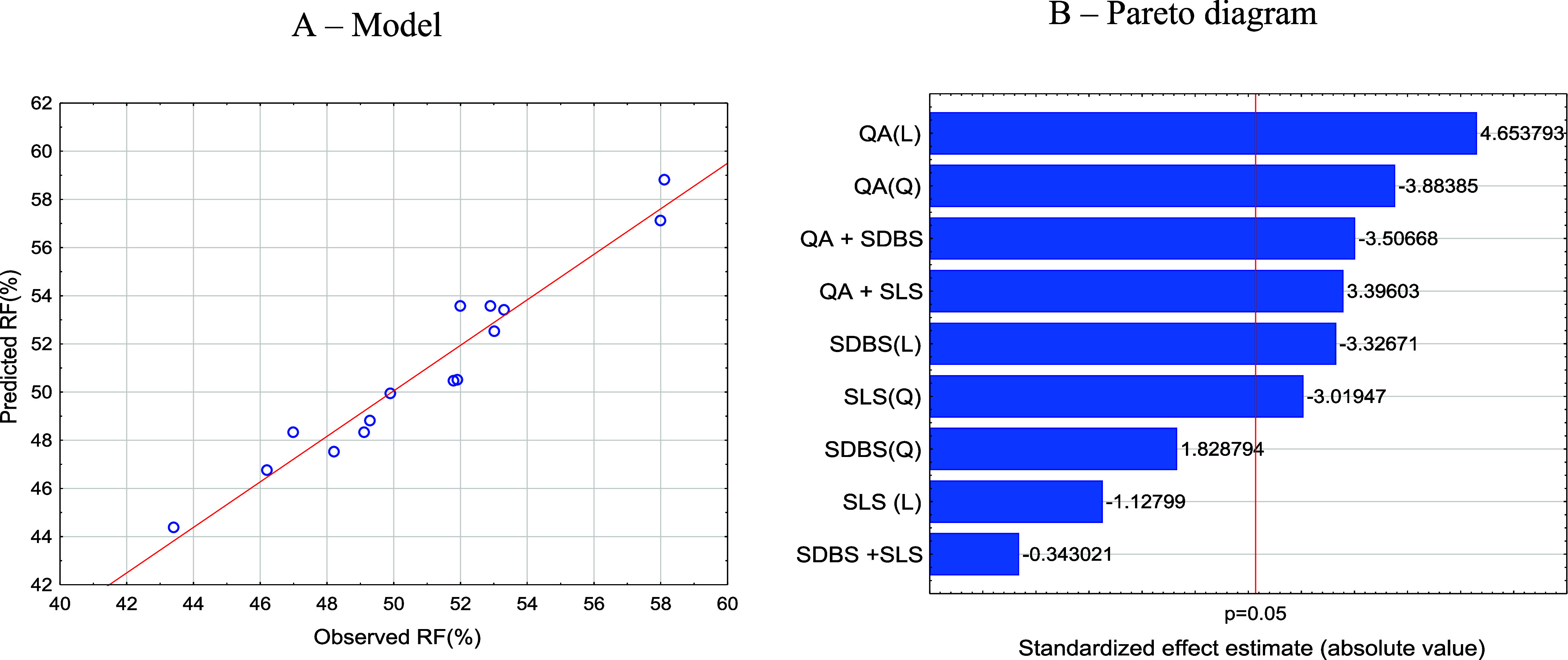
Modeling of the recovery factor (RF) of the saline solution with synergistic surfactants: (A) model comparing the measured RF with the RF predicted by the model; (B) Pareto diagram with the estimated effect standardized by the model showing the linear (L) and quadratic (Q) contributions for the surfactants and their synergistic associations: SLS; SDBS; and QA.

Figure 5A illustrates the relationship between the observed and predicted values by the chosen mathematical model. Figure 5B presents the Pareto diagram, allowing for the hierarchical observation of the influence of surfactant concentrations on the RF.

The concentration of QA demonstrated the highest statistical significance as an independent variable, exhibiting a positive linear effect of over 4.6 and a negative quadratic effect below −3.9. The dominance of the linear effect aligns with the existing literature concerning cationic surfactants with alkyl chains longer than QA.^12,21,22^ The cationic surfactant QA, composed of four small alkyl chains (two carbons each), allows for effective interaction not only with the oil on the rock surface but also due to its cationic nature; it has strong interactions with the acidic crude oil found in pre-salt formations, resulting in simultaneous effects.

For SLS (Figure 5B), with concentrations above the CMC (micellar form), both linear and quadratic contributions are negative, below −1.1 and −3.3, respectively. Additionally, it is observed that the linear contribution is below the 5% *t*-test.

In the case of SDBS (Figure 5B), with concentrations below the CMC (in the alkyl form), its linear contribution to the RF is negative below −3.3, and its quadratic contribution is positive above 1.8. However, the quadratic contribution is already below the 5% *t*-test margin.

The synergy between the surfactants QA and SLS can be observed in Figure 5B. There is a positive synergy above 3.395. This means that the presence of the cationic surfactant QA in its amphiphilic form and the anionic surfactant SLS in its micellar form increases the RF, which can be attributed to the nonmicellar QA displacing oil on the carbonate surface and SLS micelles mobilizing the oil.

The synergy between the surfactants QA and SDBS can be observed in Figure 5B. There is a negative synergy below −3.5. This means that the presence of the cationic surfactant QA and the nonionic surfactant SDBS in their non-micellar amphiphilic forms negatively affects the RF. This could be attributed to QA having its oil mobilization effect reduced due to interactions with the non-micellar forms of SDBS.

The synergies between the surfactants SDBS and SLS can be observed in Figure 5B. There is a small negative synergy below −0.34, below the 5% *t*-test. This means that the presence of the nonionic surfactant SDBS and the anionic surfactant SLS in their micellar form slightly reduces the RF. This reduction could be attributed to interactions between them that decrease the efficiency of oil mobilization by SLS micelles.

To elucidate trends and variations in the ionic composition of the produced water during low salinity water injection, a thorough analysis of collected samples was performed using X-ray fluorescence spectroscopy. This analytical approach provided valuable insights into the elemental composition of the produced water. Subsequently, the acquired data underwent multivariate statistical analysis using principal component analysis (PCA) and partial least squares (PLS).

For PCA, the general matrix was centered around the mean and principal components (PCs) 1 and 2 best separated the samples. For PLS, the relationship between the injected pore volume and the spectra obtained from the produced water analysis was modeled.

**Figure 6:**
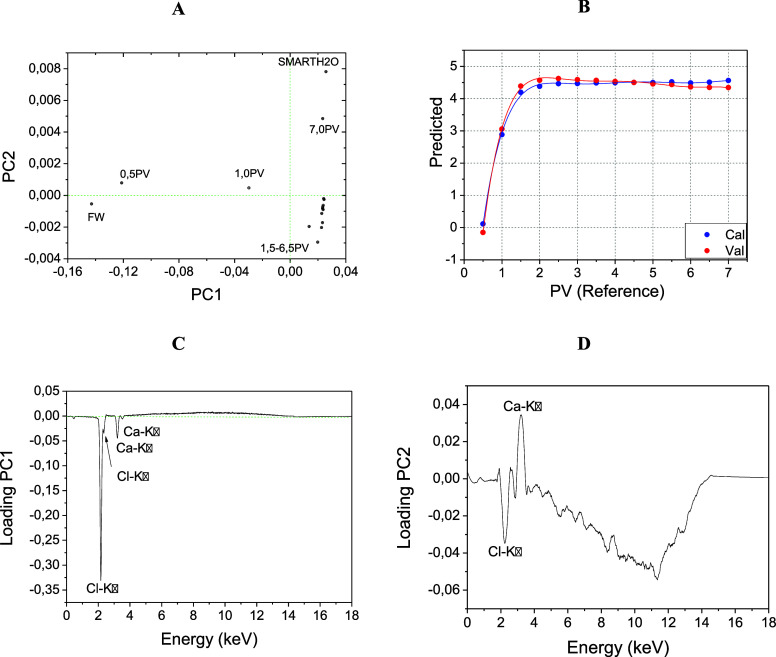
PCA and PLS analyses applied to the X-ray fluorescence spectra of the produced water for the low salinity water that yielded the highest RF (58.1%): SLS 500 ppm; SDBS zero; QA 80 ppm—RF = 58,1%. (A) PC1 versus PC2; (B) PLS model; (C) PC1 loadings; (D) PC2 loadings.

Figure 6 illustrates the outcomes, showcasing the PCA and PLS analyses’ results derived from the X-ray fluorescence spectra of the produced water, specifically from the low salinity water formulation that yielded the highest RF (58.1%): SLS 500 ppm; SDBS zero; QA 80 ppm.

PC1 and PC2 (Figure 6A) collectively accounted for 73% of the variance. In the partial least squares (PLS) modeling results (Figure 6D), the correlation between actual and predicted values showed RMSEC = 0.018 and *R*^2^ = 0.998, demonstrating exceptional performance with a 6° polynomial behavior. This high level of accuracy and sensitivity remained consistent across all tested low salinity waters.

Based on their loadings, the trend indicates that as PC1 becomes more negative (Figure 6C), the chlorine contribution predominates over calcium. For PC2 (Figure 6D), a negative semiaxis signifies higher chlorine levels, while a positive semiaxis signifies higher calcium levels.

Upon examination of Figure 6A, it is evident that the formation water (fw), containing NaCl and CaCl_2_, demonstrates a low PC1 due to its high chlorine concentration. In contrast, the low salinity water exhibits high PC2 values, signaling its elevated calcium concentration owing to the presence of approximately 2,000 ppm of CaCl_2_.

With each subsequent injection of porous volume, the divergence between the produced water samples and the formation water becomes more pronounced, leading to an increase in their PC1 value. This rise in PC1 signifies a decrease in chlorine concentration. Meanwhile, PC2 remains relatively steady around zero until the injection of 1.0 porous volumes, after which it shifts to negative values, indicating an escalation in the chlorine atom concentration. Upon injection of seven porous volumes, a strong increase in calcium is observed, implying that the formation water may have inherently contained more calcium, likely due to interactions with the limestone surface.

**Figure 7:**
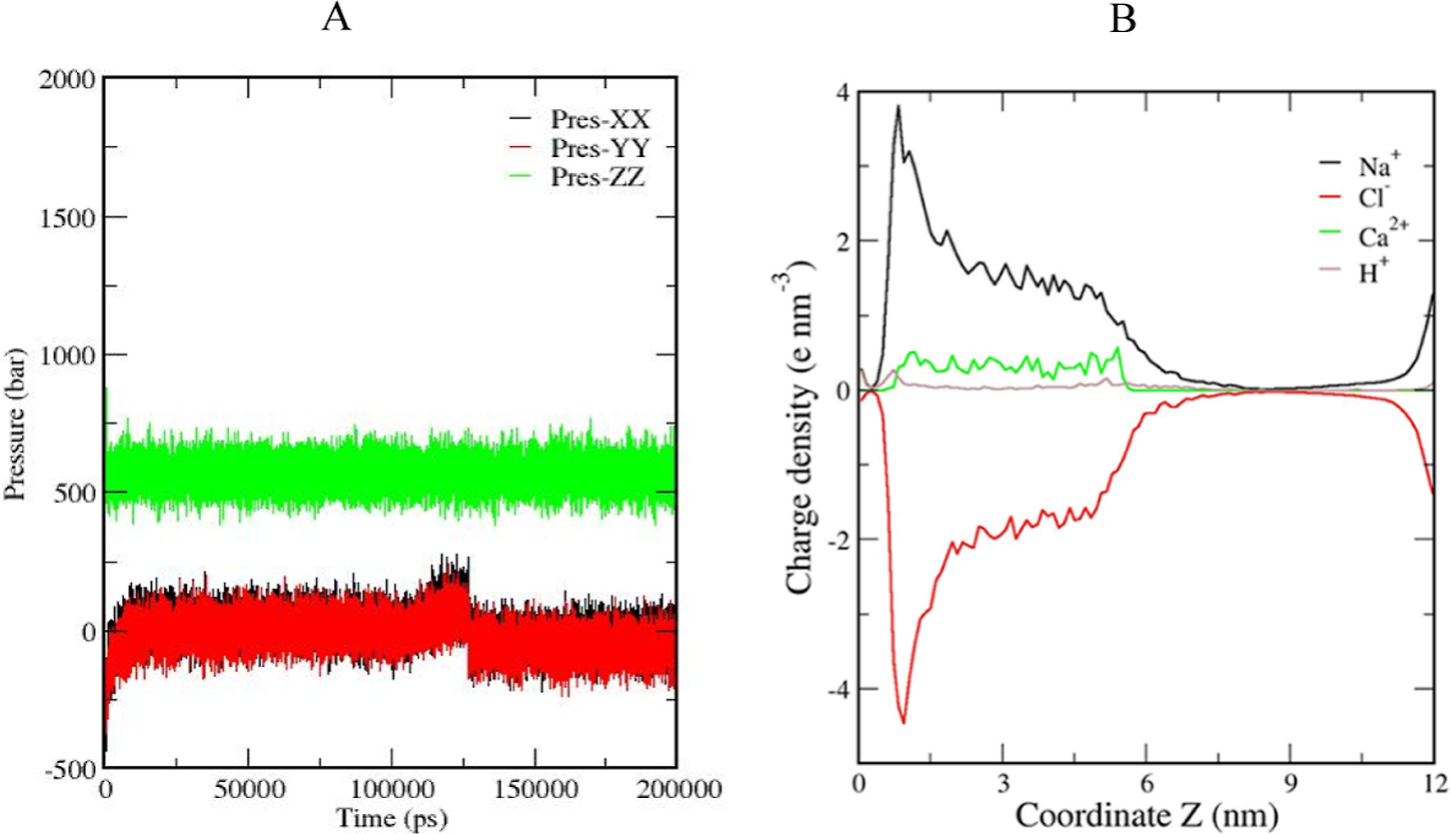
(A) Evolution of the pressure components (eq 1) during the simulation; (B) charge density distribution applied to the surface of calcite.

In the first theoretical simulation, SLS (80 ppm) and QA (500 ppm) surfactants were added to synthetic brine containing NaCl and CaCl_2_. Figure 7A shows the pressure component curves used to estimate the interfacial tensions. Figure 7B indicates that changes in ion concentration on the rock surface cause it to become slightly positive due to the accumulation of Na^+^ ions. The surface charge on the rock can be assessed through the charge density distribution, yielding a value of 0.15 e nm^2^, indicating a more positively charged surface. This surface charge reduces the diffuse double layer, resulting in a brine density of around 1.09 g/mL. This value is very close to that measured for synthetic formation water mixed with deionized water.

**Figure 8:**
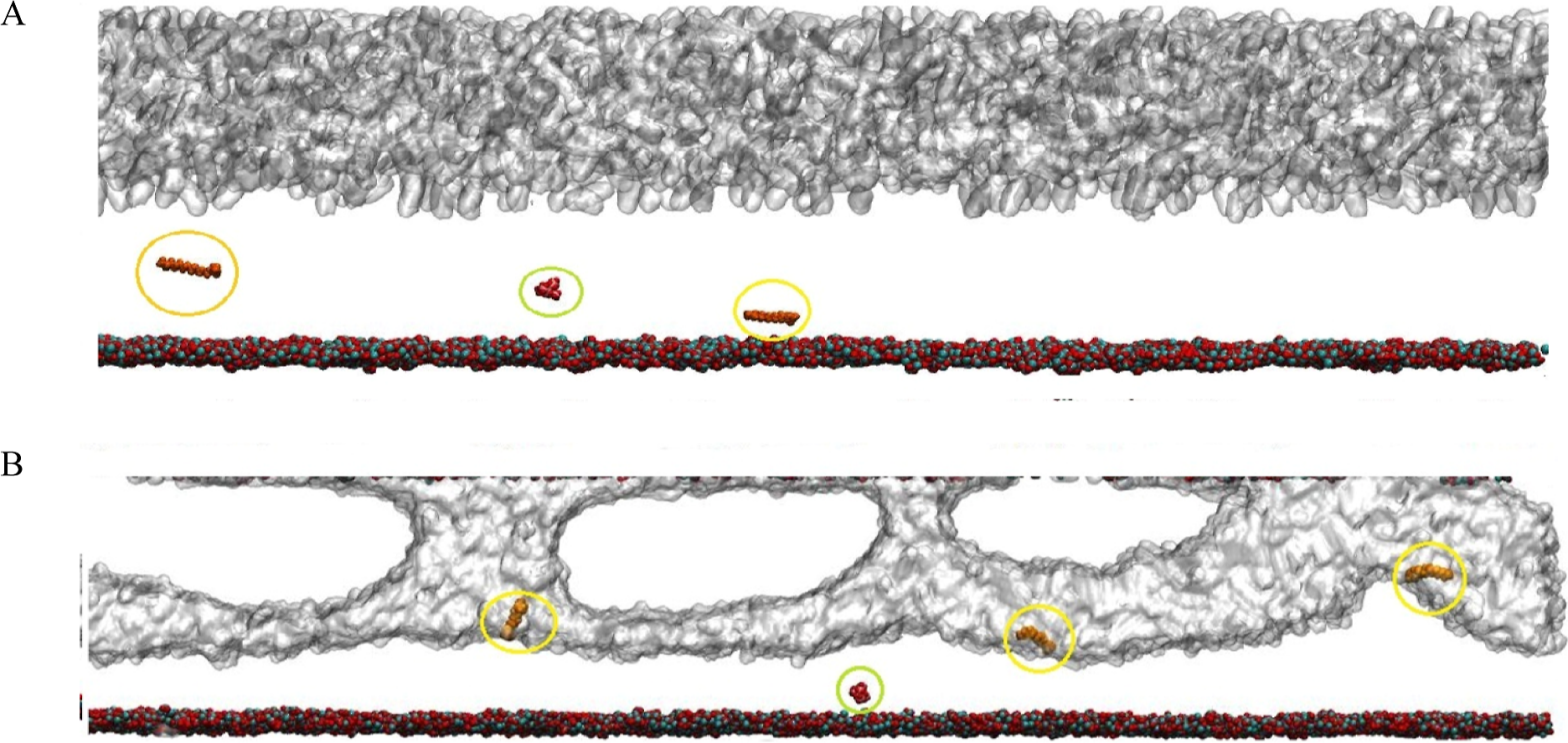
Theoretical simulation with SLS highlighted by a yellow circle and QA by a green circle; oil is gray and rock is cyan/red. For clarity, the brine is not shown. (A) Initial configuration, where the surfactants were injected with low salinity water. (B) Final configuration, showing the SLS molecules at the oil/brine interface.

During the simulation, SLS molecules migrated toward the oil–brine interface, interacting with Na^+^ ions, while QA remained in the brine bulk, as shown in Figure 8. Due to the immobilization of surfactants in specific regions of the simulation box, nonspecific interactions (electrostatic and van der Waals) resulted in a null value, indicating that under these conditions, the surfactants did not interact with each other.

The Einstein relation was applied to calculate viscosity by integrating the correlation function, yielding a calculated viscosity of 0.66 cP for the brine at the given concentration. This result aligns closely with the measured value of 0.72 cP, with the slight discrepancy potentially attributable to the presence of NaHCO_3_. Since most SLS molecules are located at the liquid–liquid interface, the interfacial tension (IFT) is estimated to be 51.75 mN/m.

In a second simulation, surfactants were used at concentrations of SBDS (800 ppm), QA (80 ppm), and SLS (500 ppm), resulting in a computed interfacial tension (IFT) of 64.68 mN/m. Although the increased total surfactant concentration, with the addition of SBDS, suggests a response in the increase in brine viscosity compared with the value calculated in the first simulation, it is expected to negatively impact the RF, as observed in Table 3.

**Table 3:** Relative Energy Between Surfactants

energy (kJ/mol)	QA-SDBS	QA-SLS	SLS-SDBS
electrostatic	–139.67 ± 0.17	0.00	–187.25 ± 0.32
van der Waals	11362.46 ± 2.31	0.00	208825.40 ± 4.79

Table 3 suggests a strong repulsion between surfactants, as indicated by the van der Waals term. This implies that the surfactants do not interact with each other. However, this combination increases the viscosity of the brine (0.95 cP). The results suggest an additive effect of the tested surfactants on viscosity while also pointing to an increase in interfacial tension (IFT).

## 4 Conclusions

This study aimed to develop smart water EOR formulations customized for limestone reservoirs aligning with the United Nations Sustainable Development Goal (SDG) 12 and the 2030 Agenda. The focus was on minimizing the environmental impact through the use of surfactants with low concentrations, contributing to a responsible energy transition.

The linear analysis of individual surfactant concentration variations yielded an increase in the RF of up to 12.8%. Nevertheless, employing a covariation strategy resulted in additional enhancements, elevating the RF by up to 16.7%. This improvement was primarily attributed to the synergistic interaction between the cationic and anionic surfactants, optimizing the balance among all of the concentration components.

The most promising outcomes resulted from the synergistic combinations of non-micellar QA and micellar SLS at lower concentrations. The modeling suggested a potential increase in RF by elevating the concentration of the non-cationic surfactant. Experimentally, this increased the RF by approximately 0.8%. However, since it contradicted the study’s environmental objectives by potentially escalating environmental impact, this approach was not pursued. Theoretical simulations corroborated the experimental findings, aligning with the observed results.

Thus, it became feasible to achieve higher increments in RF without resorting to the typical high concentrations of surfactants. This was accomplished by maximizing the synergistic effects of cationic, anionic, and nonionic surfactants, along with their respective micellar and non-micellar forms. These outcomes contribute to ensuring that given the continued necessity of fossil fuels for decades to support the responsible energy transition, it is possible to attain a substantial EOR RF with minimal environmental impact potential.
